# Sex differences in conduct and emotional outcomes for young people with hyperactive/inattentive traits and social communication difficulties between 9 and 16 years of age: a growth curve analysis

**DOI:** 10.1017/S0033291722001416

**Published:** 2023-07

**Authors:** Jack Hollingdale, Emma Woodhouse, Susan Young, Gisli Gudjonsson, Tony Charman, Will Mandy

**Affiliations:** 1 University College London, London, UK; 2 King's College London, London, UK; 3 Psychology Services Ltd, London, UK

**Keywords:** Adolescence, ALSPAC, attention deficit hyperactivity disorder, autism spectrum disorder, children, conduct problems, emotional problems, social communication difficulties, young people

## Abstract

**Background:**

The purpose of this paper is to identify the trajectory of conduct and emotional problems for young people within the general population at four time points (between 9 years 7 months and 16 years 6 months), investigate their relationship with hyperactive/inattentive traits and explore the moderating effect of autistic social traits (ASTs).

**Methods:**

Data from 9305 individuals involved in The Avon Longitudinal Study of Parents and Children (ALSPAC) study were included. Conduct and emotional problems and hyperactive/inattentive traits were measured by the Strengths and Difficulties Questionnaire. ASTs were assessed using the Social Communication Disorder Checklist. Individual trajectories for conduct and emotional problems were identified via growth curve modelling. Hyperactive/inattentive traits were included within the growth curve model as a time-varying covariate to determine their effect on these outcomes. Finally, participants were split into two groups (below and above clinical threshold ASTs Groups) and multi-group invariance testing was conducted on the data to identify the moderating effect of ASTs on the relationship between hyperactive/inattentive traits and outcomes (i.e. conduct and emotional problems).

**Results:**

Hyperactive/inattentive traits were associated with higher rates of conduct and emotional problems for both boys and girls. The presence of ASTs moderated these relationships for boys, but not for girls, by increasing the risk of boys with hyperactive/inattentive traits developing greater conduct and emotional problems.

**Conclusions:**

These findings underscore the importance of identifying hyperactive/inattentive traits and ASTs in young people and addressing the increased risk of conduct and emotional problems. Research and clinical implications are explored.

## Introduction

Attention deficit hyperactivity disorder (ADHD) is one of the most common psychiatric conditions experienced by young people (defined here as males and females under 18 years of age) and is associated with a range of co-occurring conditions (American Psychiatric Association (APA), 2013). This includes conduct problems and emotional difficulties, which cause functional impairments across different life domains (Deault, 2010).

High rates of co-occurring neurodevelopmental atypicalities, specifically autistic social traits (ASTs), have been identified in young people with ADHD (Hollingdale, Woodhouse, Young, Fridman, & Mandy, 2020; Larson, Russ, Kahn, & Halfon, 2011; Pourcain et al., 2011) and are also associated with conduct and emotional problems (Mandy, Skuse, Steer, St Pourcain, & Oliver, 2013; Oliver, Barker, Mandy, Skuse, & Maughan, 2011). Therefore, the presence of both ADHD traits and ASTs may exacerbate the frequency and severity of difficulties.

The current research will explore the relationships between ADHD traits and conduct/emotional problems over time. Due to high rates of co-occurring ASTs, it will also investigate the relationship that co-occurring AST's have on these relationships.

### Attention deficit hyperactivity disorder (ADHD)

ADHD is a neurodevelopmental disorder characterised by a persistent pattern of inattention and/or hyperactivity-impulsivity which impairs daily living or typical development (APA, 2013). The ADHD worldwide-pooled prevalence is 5.29–7.2% (Polanczyk, De Lima, Horta, Biederman, & Rohde, 2007; Thomas, Sanders, Doust, Beller, & Glasziou, 2015) and rates are comparable across cultures (Faraone, Sergeant, Gillberg, & Biederman, 2003). The profile of ADHD symptoms is known to change. Both hyperactive/impulsive and inattentive symptoms can reduce over time (Francx et al., 2015; Willcutt et al., 2012). However, this may partially be attributed to developing management strategies with age.

ADHD is increasingly being conceptualised dimensionally rather than categorically (Coghill & Sonuga-Barke, 2012; Frazier, Youngstrom, & Naugle, 2007; Lubke, Hudziak, Derks, van Bijsterveldt, & Boomsma, 2009; Marcus & Barry, 2011; Nigg, Goldsmith, & Sachek, 2004; Polderman et al., 2007; Sonuga-Barke, 2005). This change in thinking has resulted from similar rates of heritability between those with low, moderate and high rates of attentional difficulties (Gjone, Stevenson, & Sundet, 1996), recognition of fluidity of symptoms between ADHD categories (Lahey, Pelham, Loney, Lee, & Willcutt, 2005) and a greater emphasis on the severity of impairment rather than symptoms (Haslam et al., 2006).

Sex differences have been identified between males and females with ADHD. Community and clinical referral rates for ADHD diagnoses are higher for males than females, with ratios between 3:1 and 16:1 (Nøvik et al., 2006; Willcutt, 2012), suggesting possible biases in the identification of the condition for males. Higher rates in males have been linked to increased genetic vulnerability and differences in psychosocial factors (Hinshaw, 2018; Zuddas, Banaschewski, Coghill, & Stein, 2018). In addition, females with ADHD differ from males in their symptom profile, co-occurring conditions and functional impairments (Biederman et al., 2005; Young et al., 2020a).

The onset of ADHD is different for each individual and the age range during which ADHD symptoms manifest is debated in the literature (Kieling et al., 2010). However, as ADHD is a neurodevelopmental condition, ADHD symptoms can often proceed conduct and emotional problems (Loeber & Hay, 1997; Loeber, Green, Keenan, & Lahey, 1995; Stern et al., 2020). The precise genetic, psycho-biological, environmental and contextual mechanisms for this are beyond the scope of this paper.

### Conduct and emotional problems

Conduct problems (also known as ‘externalising problems’) are actions carried out in the external world, such as antisocial behaviour, hostility, aggression and substance misuse (McMahon, Wells, & Kotler, 2006). However, some conduct problems such as alcohol misuse may arise as a consequence of emotional problems (Rosenfield, Lennon, & Raskin White, 2005). Emotional problems (also known as ‘internalising problems’) are characterised as internal processes, such as anxiety or depression (Forms, Abad, & Kirchner, 2011). Due to their internal manifestation, they are often more difficult to identify than conduct problems.

The prevalence and trajectories of conduct and emotional difficulties differ between males and females. Boys are reported to experience more conduct difficulties than girls (Broidy et al., 2003). Despite some variation, this pattern persists across different ethnic groups (McLaughlin, Hilt, & Nolen-Hoeksema, 2007). Over time, conduct problems reduce for both boys and girls, but emotional problems increase for girls (Leve, Kim, & Pears, 2005).

### Conduct problems

#### ADHD

Research indicates that the symptom overlap between ADHD and conduct disorder is partially due to shared genetic aetiology between the conditions (Faraone, Biederman, Mennin, Russell, & Tsuang, 1998). As many as 30% of young people with ADHD in the general population meet diagnostic criteria for conduct disorder (Wolraich, Hannah, Baumgaertel, & Feurer, 1998) and up to 50% in clinical samples (Newcorn et al., 2001). Rates of co-occurrence are lower for females (Biederman et al., 2002). Research has identified that sub-diagnostic ADHD traits predict associated features of ADHD, including conduct problems and emotional problems (Marcus & Barry, 2011). However, further research is required to understand the relationship between ADHD traits and conduct problems between males and females.

Familial environments also affect the association between ADHD and conduct problems. For example, conflict within the family moderates the association between ADHD and conduct problems for both boys and girls. (Sigfusdottir et al., 2017).

Previous research has shown an inconsistent relationship between ADHD, oppositional defiant disorder (ODD) and conduct disorder for both males and females (Costello, Mustillo, Erkanli, Keeler, & Angold, 2003; Lahey, McBurnett, & Loeber, 2000). However, more recent research has identified that ADHD predicts ODD and conduct disorder for both sexes (Bendiksen et al., 2017; Ottosen et al., 2019).

#### Social communication difficulties

Social communication difficulties include deficits in social reciprocity and verbal/nonverbal abilities which are core diagnostic features of autism spectrum disorder (ASD) (autism) (APA, 2013). Irrespective of diagnostic thresholds, elevated social communication difficulties are associated with functional impairments, specifically behavioural difficulties (Hoch & Symons, 2007) and conduct problems (Skuse et al., 2009).

Deficits in social-cognition (the cognitive ability to respond appropriately during social interactions) have been linked to conduct problems (Oliver et al., 2011) and ASTs moderate the relationship between oppositionality and conduct problems in mid-and- late childhood (Mandy et al., 2013).

### Emotional problems

#### ADHD

Young people with ADHD, and particularly females, are more likely to experience emotional difficulties (Rapee et al., 2019; Schatz & Rostain, 2006) and are more likely to receive a diagnosis of Generalised Anxiety Disorder (Gershon & Gershon, 2002; Safren, Lanka, Otto, & Pollack, 2001). Young people with ADHD are also more likely to experience depression and higher rates of depression are associated with higher rates of anxiety (Blackman, Ostrander, & Herman, 2005). These findings are indicative of a complex and multidirectional relationship between ADHD and emotional problems and the co-occurrence of mental health conditions increases the risk of long-term impairments and suicide (Daviss, 2008).

#### Social communication difficulties

Autistic young people are more likely to experience anxiety, depression and mood problems than their non-autistic peers (Kanne, Abbacchi, & Constantino, 2009; Kim, Szatmari, Bryson, Streiner, & Wilson, 2000; Leyfer et al., 2006; Simonoff et al., 2008; White, Oswald, Ollendick, & Scahill, 2009). Young people with autism-like traits (but are below the diagnostic threshold for autism) also experience greater anxiety and depression than young people with fewer autism-like traits (Lundström et al., 2011). Specifically, young people with more ASTs experience greater social anxiety (Pickard, Rijsdijk, Happé, & Mandy, 2017; Skuse et al., 2009) than those with fewer ASTs. In addition, autistic females are more likely to experience anxiety, depression and somatic symptoms than autistic males (Hatta, Hosozawa, Tanaka, & Shimizu, 2019; Solomon Miller, Taylor, Hinshaw, & Carter, 2012).

#### ADHD and Co-occurring social communication difficulties

An estimated 21% of young people with ADHD experience co-occurring autism (Hollingdale et al., 2020) and significant correlations have been found between ADHD and autistic traits within the general population (Hollingdale et al., 2020; Ronald, Simonoff, Kuntsi, Asherson, & Plomin, 2008).

Co-occurring ADHD and autism is associated with higher rates of general psychopathology compared with having one diagnosis but not the other (Holtmann, Bölte & Poustka, 2007). For example, there are higher rates of cognitive impairment (Rao & Landa, 2014), including verbal memory, recall (Andersen, Hovik, Skogli, Egeland, & Øie, 2013), sustained attention (Sinzig, Bruning, Morsch, & Lehmkuhl, 2008), social impairment (Rao & Landa, 2014), adaptive behaviour (Yerys et al., 2009), and oppositional defiant disorder and conduct disorder (Mulligan et al., 2009). Young people with both ADHD and autism experience higher rates of oppositionality and aggression, tantrum behaviours, conduct problems, worry and depression than young people who have ADHD or autism without co-occurring conditions (Guttmann-Steinmetz, Gadow, & DeVincent, 2009; Guttmann-Steinmetz, Gadow, DeVincent, & Crowell, 2010; Jang et al., 2013). Furthermore, research indicates that young people with both ADHD and autism have greater adaptive functioning difficulties and a poorer health-related quality of life than their peers without these conditions. (Sikora, Vora, Coury, & Rosenberg, 2012). Due to the limited number of females included in previous studies investigating co-occurring ADHD and autism, it is difficult to ascertain whether difficulties manifest differently for males and females.

It is important to acknowledge there may be sex differences in the presentation of ADHD and autism. For ADHD, girls may experience lower levels of hyperactivity, impulsivity and (externalised) behavioural problems than boys. They may also experience higher rates of (internalising) emotional problems (Gaub & Carlson, 1997; Gershon & Gershon, 2002). Autistic girls may have more severe communicational difficulties, anxiety, depression (Hartley & Sikora, 2009) and behavioural problems compared with boys (Dworzynski, Ronald, Bolton, & Happé, 2012).

#### Aims of the current study

There are reported sex differences in the presentation of ADHD and autism. For ADHD, girls may experience lower levels of hyperactivity, impulsivity and (externalised) behavioural problems than boys. They may also experience higher rates of (internalising) emotional problems (Gaub & Carlson, 1997; Gershon & Gershon, 2002). Autistic girls may have more severe communicational difficulties, anxiety, depression (Hartley & Sikora, 2009) and behavioural problems compared with boys (Dworzynski et al., 2012). Despite emerging evidence for the relationship between conduct problems and ADHD (Sigfusdottir et al., 2017), further research is required to assess whether ADHD (across the full spectrum of severity) is associated with higher conduct and emotional problems.

Current literature lacks clarity about the extent to which ASTs affect the relationship between ADHD traits and conduct/emotional problems. To our knowledge, this has not been previously investigated comparing boys and girls using a longitudinal population-based methodology.

The current study aimed to investigate the extent to which dimensionally-measured ADHD traits are associated with conduct and emotional problems for boys and girls within the general population; and the extent to which dimensionally-measured ASTs moderate this relationship. To this end, the current study addresses the following questions:

1. What association is there between hyperactive/inattentive traits and conduct/emotional problems for boys and girls between the ages of 9 and 16?
2. Do ASTs moderate the conduct and emotional problem trajectories for boys and girls differently?

## Method

### Design of original study

The Avon Longitudinal Study of Parents and Children (ALSPAC) is a longitudinal general population cohort measuring the health and development of young people. Pregnant women resident in Avon, UK, with expected dates of delivery 1 April 1991 to 31 December 1992 were eligible for participation. The final cohort consisted of 15 454 pregnancies of which 14 901 births were alive at one year of age (Boyd et al., 2013; Fraser et al., 2012).

Please note that the study website contains details of all the data that is available through a fully searchable data dictionary and variable search tool (http://www.bristol.ac.uk/alspac/researchers/our-data/).

### Present study

#### Participants

Participants' trait data were extracted at four time points: Time 1 (average age 9 years and 7 months), Time 2 (11 years and 8 months), Time 3 (13 years and 1 month), and Time 4 (16 years and 6 months). Participants were included in the study if they had completed data from at least one time point.

Of the 9305 young people included within this study (boys = 4675 and girls = 4630), 49.8% were female, 96.1% were white, 15.5% had mothers with a university degree, and 81.1% had a parent who owned their own home (see Table 1). There were no statistically significant sex differences for these demographics. Only those who completed the relevant measures were included within the current study. Compared with participants of the original cohort who were not included within this analysis, the current participants were more likely to have a mother who: had a degree (OR = 2.40, 95% CI [2.09, 2.74]); and was a home owner (OR = 2.93, 95% CI [2.71, 3.17]). Compared to those included within the study, those who were not included were more likely to be from a minority ethnic background (OR 1.99, 1.69, 2.34).

**Table 1.** Differences between those included and excluded from analysis

	Included		Excluded	
	Male	Female	Male	Female
Sex^a^	4675	4630	2960	2589
	Yes	No	Yes	No
Mother has degree	1330	8573	279	3920
Mother is home owner	6989	1633	2897	1982
Child from non-white ethnic background	332	8091	281	3447

a Data missing for 591 young people.

#### Measures

*The Strengths and Difficulties Questionnaire* (*SDQ*): The SDQ is a 25-item behavioural screening questionnaire which is divided into five scales: internalising difficulties, conduct problems, hyperactivity/inattention traits, peer relationship problems and prosocial behaviour. Parents are asked to consider their child's behaviour in the last six months, using a three-point Likert scale, and identify the extent to which each attribute applies to their child; ‘not true’, ‘somewhat true’ and ‘certainly true’.

The SDQ is a valid screening tool for multi-dimensional behaviour and mental health in the general population (Goodman, 2001; Goodman & Goodman, 2009). Research indicates that it is an effective dimensional measure of psychopathology (Goodman & Goodman, 2011) within clinical populations (Becker, Woerner, Hasselhorn, Banaschewski, & Rothenberger, 2004) and across different cultural and ethnic populations (Mieloo et al., 2013; Muris, Meesters, & van den Berg, 2003).

Croft, Stride, Maughan, and Rowe (2015) identified moderate to good internal reliability and construct validity for each of the five scales of the SDQ for young people and importantly that the item-factor structures did not change over time.

*The Social Communication Disorder Checklist* (*SCDC*): The SCDC is 12-item scale which requires parents to rate their child's social reciprocity and verbal/nonverbal characteristics during the previous six months. However, it should be noted that the SCDC does not investigate the restricted and repetitive behaviours and interests associated with ASD, and therefore this paper focuses on the social reciprocity and social communication difficulties associated with ASD (i.e. ASTs). Items are scored 0 (‘not true’), 1 (‘quite or sometimes true’) and 2 (‘very or often true’). Thus, scores range from 0 to 24 with higher scores reflecting greater difficulties with social communication. Total scores of eight or greater are considered to be clinically significant (Skuse et al., 2009).

The SCDC is an effective first-level screening questionnaire and dimensional measure of ASTs (Skuse, Mandy, & Scourfield, 2005). Skuse et al. (2005) found that traits measured by the SCDC were highly heritable in both sexes (0.74). Internal consistency was excellent (0.93) and test–retest reliability was high (0.81). Discriminant validity between pervasive developmental disorder and other clinical groups was good, discrimination from non-clinical samples was better; sensitivity (0.90), specificity (0.69). Further evidence of construct validity comes from the findings that variability in SCDC scores is partly driven by genetic effects that also influence risk of a clinical autism diagnosis (Robinson et al., 2016).

#### Procedure

Data from the SDQ was extracted at four time points and the SCDC data was recorded when participants were aged 7 years and 8 months because autistic traits, as measured by the SCDC, have been reported to remain highly stable over time (Pourcain et al., 2011; Robinson et al., 2011).

#### Analyses

*Research Question 1: What association is there between hyperactive/inattentive traits and conduct/emotional problems for boys and girls between the ages of 9 and 16?*

Trait trajectories of conduct and emotional problems were identified as a precursor to investigating the relationship of hyperactivity/inattention and these outcomes. Item-level missing data were addressed by using proration in accordance with recommendations by Graham (2009). Thus, for participants with 50% or less item-level missing data the mean of a participant's observed scores was imputed into their missing scores prior to the conduction of any analysis. Separate growth curve models were then built to characterise change in conduct and emotional problems between the ages of 9 and 16 for all participants.

All models were constructed in Analysis of Moment Structures (AMOS) Version 24 (Arbuckle, 2016) and were estimated using a maximum likelihood estimator (MLE). MLE is the most popular and recommended method of estimation, to deal with missing data, due to being a totally analytical maximisation procedure (Scholz, 2014).

Growth curve modelling was used to identify trajectories for conduct and emotional problems over time. Initially, linear growth curve models were built for all 9305 participants. Model fit was evaluated using the χ^2^ goodness-of-fit test, comparative fit index (CFI), root mean square error of approximation (RMSEA) and Akaike's information criterion (AIC). A CFI value of 0.90 or greater was considered an acceptable fit (Bentler, 1992) and a value of 0.95 or greater was considered to represent a good fitting model (Hu & Bentler, 1999). RMSEA values less than 0.05 indicated good fit, and between 0.05 and 0.08 reasonable fit (Browne & Cudeck, 1993). Values between 0.08 and 0.10 indicated mediocre fit and greater than 0.10 represented a poor-fitting model (MacCallum, Browne, & Sugawara, 1996). Regarding the AIC, when comparisons are made between two models, smaller values represented a better fitting model (Hu & Bentler, 1995). In addition, quadratic terms were included across models to account for the non-linear change. To determine whether trait trajectories differed between males and females, sex was included within the conduct and emotional problems models as a predictor variable, see online Supplementary Materials for means and standard deviations of outcomes by gender and time points.

A univariate growth model with time-varying covariates was used to determine whether hyperactive/inattentive traits affect the trajectories of conduct and emotional problems (Muthén & Muthén, 2009). Time-varying covariates allow for the values of the covariate to change over time, in this case hyperactive/inattentive traits, and have an effect on the outcome variables; conduct and emotional problems (Muthén, 2002). The inclusion of hyperactive/inattentive traits as a time-varying covariate determined whether hyperactive/inattentive traits are associated with conduct and emotional problems at each time point, above and beyond the trajectory processes underlying the development of these problems. In other words, whether hyperactive/inattentive traits are associated with variability in the trajectory of conduct and emotional problems over time. Although the value of the time-varying covariate can change across time, the parameter value estimating the effect of the time-varying covariate on the outcomes are assumed to be constant across time (McCoach & Kaniskan, 2010).

*Research Question 2: Do ASTs moderate the conduct and emotional problem trajectories for boys and girls differently?*

To determine whether ASTs moderate the relationship between hyperactive/inattentive traits and conduct and emotional problems it was necessary to identify whether these relationships were different between young people who were reported to have fewer or more ASTs. To achieve this, multi-group invariance testing was carried out (i.e. testing for sameness or non-change).

Boys and girls were grouped by their total SCDC scores: Below clinical threshold (scores equal to or less than seven) and Above clinical threshold (scores equal to or greater than eight) (Skuse et al., 2009). Our multi-group approach tested whether the relationships between hyperactive/inattentive traits and the outcome variables (i.e. conduct problems, emotional problems) at each time point were different for the Below and Above Group.

## Results

*Research Question 1: What is the relationship between hyperactive/inattentive traits, conduct and emotional problems for boys and girls between the ages of 9 and 16?*

In all cases, models that included a quadratic term best fit the data for conduct and emotional problems, see online Supplementary Materials. Therefore, a quadratic term was included in all further models (Byrne, 2013). Good fitting models were identified for both boys and girls when hyperactive/inattentive traits were added as a time-varying covariate at each time point, see online Supplementary Materials.

Results indicated that both boys and girls with greater hyperactive/inattentive traits had significantly greater conduct and emotional problems at all ages than would be expected by their individual trajectories alone (*p* < 0.001), see Table 2.

**Table 2.** Standardised regression weights (*β*) of hyperactive/inattentive traits on conduct problems (CP) and emotional problems (EP) for Model 1

	Boys		Girls	
	CP (*N* = 4675)	EP (*N* = 4671)	CP (*N* = 4630)	EP (*N* = 4629)
H/I Time 1	0.282*	0.205*	0.244*	0.194*
H/I Time 2	0.270*	0.231*	0.254*	0.223*
H/I Time 3	0.275*	0.216*	0.268*	0.239*
H/I Time 4	0.263*	0.224*	0.292*	0.344*

* *p* < 0.001.

*Research Question 2: Do ASTs moderate the conduct and emotional problem trajectories for boys and girls differentially?*

Applying the clinical threshold cut-off of 8, the boys and girls were divided into either a Below Group or Above Group, see Table 3 for group *n*'s. Multi-group invariance testing was conducted for boys and girls separately.

**Table 3.** Group *n*'s by gender and conduct and emotional problems

	Boys		Girls	
	Conduct	Emotional	Conduct	Emotional
Below Group	3317	3314	3394	3393
Above Group	458	459	251	250
Total	3775	3773	3645	3643

For girls, invariance was identified between the Below and Above Groups for both conduct problems (Δχ^2^ = 4.842, *p* = 0.304) and emotional problems (Δχ^2^ = 2.511, *p* = 0.643). Thus, fixing the pathways from hyperactivity/inattention to the outcome variables at all four time points did not reduce model fit. This indicates that ASTs do not moderate the relationship between hyperactive/inattentive traits and either conduct and emotional problems for girls across these time points.

For boys, a different pattern of results was observed. Non-invariance was identified between the Below and Above Groups in boys for both conduct problems (Δχ^2^ = 14.670, *p* < 0.01) and emotional problems (Δχ^2^ = 19.162, *p* = 0.001). Thus, there is a different effect of hyperactive/inattentive traits on conduct and emotional problems in boys with fewer ASTs and boys with more ASTs. See Table 4 for regression estimates for boys and online Supplementary Materials for regression estimates for girls.

**Table 4.** Standardised regression weights (*β*) for Below clinical threshold ASTs Group and Above clinical threshold ASTs Group for conduct problems (CP) and emotional problems (EP) for boys

	Below Group			Above Group		
	*β*	s.e.	C.R	*β*	s.e.	C.R
Conduct Problems						
H/I Time 1 ⩾ CP Time 1	0.222*	0.009	25.170	0.282*	0.030	9.254
H/I Time 2 ⩾ CP Time 2	0.222*	0.008	28.878	0.295*	0.026	11.395
H/I Time 3 ⩾ CP Time 3	0.234*	0.008	29.550	0.316*	0.028	11.444
H/I Time 4 ⩾ CP Time 4	0.229*	0.010	22.701	0.341*	0.035	9.654
Emotional Problems						
H/I Time 1 ⩾ EP Time 1	0.148*	0.012	12.687	0.252*	0.035	7.152
H/I Time 2 ⩾ EP Time 2	0.205*	0.010	20.925	0.238*	0.030	7.934
H/I Time 3 ⩾ EP Time 3	0.183*	0.010	18.609	0.255*	0.033	7.765
H/I Time 4 ⩾ EP Time 4	0.189*	0.013	14.539	0.290*	0.038	7.672

* *p* < 0.001.

Having determined the presence of non-invariance for boys, additional analyses were conducted to identify at which specific time points the presence of ASTs affected the relationship between hyperactive/inattentive traits and conduct and emotional problems.

**Fig. 1.**
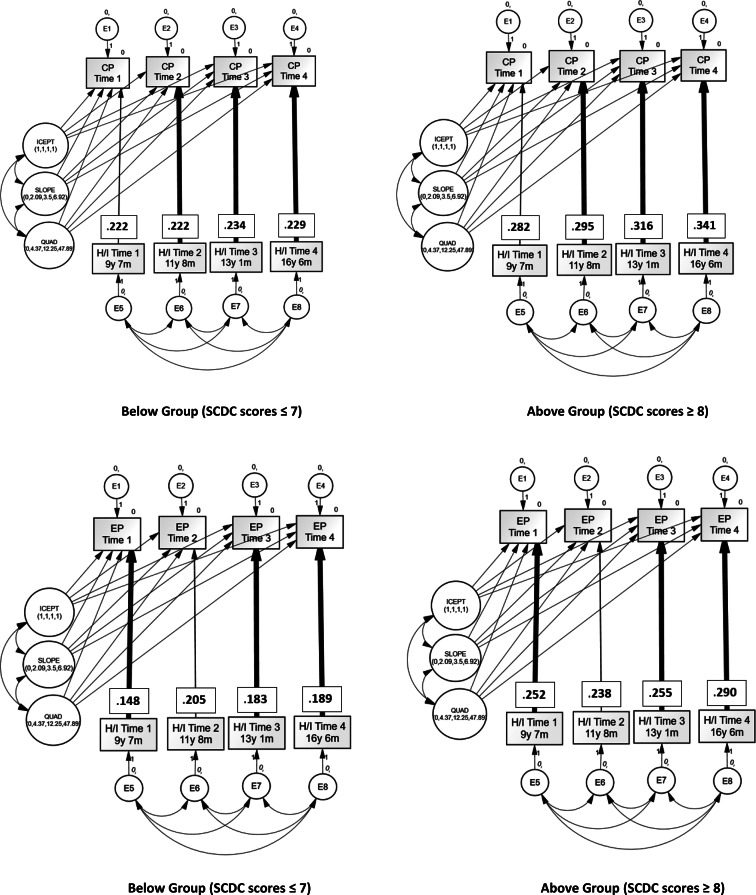
*β* regression weights of non-invariance between Below Group (left) and Above (right) for conduct problems (CP) (top) and emotional problems (EP) (bottom) for boys. Bold lines denote significantly different χ^2^ values between the two groups.

Regarding conduct problems, non-invariance was identified between the two male ASTs Groups at three time points: Time 2, Time 3, and Time 4 but not at Time 1. For emotional problems, non-invariance between the two groups was identified at Time 1, Time 3, and Time 4 but not at Time 2. See Fig. 1.

These findings of non-invariance arose because the effects of hyperactive/inattentive traits were greater for the Above Group compared with the Below Group for both conduct and emotional problems. To put this another way, the relationship between ADHD traits and conduct/emotional problems was greater for boys with elevated ASTs compared to those with lower AST's. The same did not hold true for girls.

## Discussion

The literature has consistently reported gender differences in the impact of ADHD on conduct and emotional problems for young people (Gershon & Gershon, 2002; Young et al., 2020a). This study has provided insight into this relationship and identified an important moderator in the process. Hyperactive/inattentive traits were associated with higher rates of conduct and emotional problems for both boys and girls across late childhood and adolescence. ASTs did not affect the relationship between hyperactive/inattentive traits, conduct or emotional problems in girls, but they did in boys. Hence ASTs increase the risk that boys with hyperactive/inattentive traits will experience higher levels of conduct problems, at the ages of 11, 13 and 16, and emotional problems, at the age of 9, 13 and 16. These findings offer new insights into the trajectories of these difficulties for boys and girls during childhood and adolescence.

Hyperactive/inattentive traits were associated with significantly higher levels of conduct and emotional problems for both males and females at all ages than would be expected by their individual trajectories alone. These findings extend previous research attributing increased hyperactive/inattentive trait severity to higher rates of conduct problems (Côté, Tremblay, Nagin, Zoccolillo, & Vitaro, 2002; Shaw, Lacourse, & Nagin, 2005; Waschbusch, 2002) and emotional problems (Daviss, 2008; Safren et al., 2001) over time. However, other factors, such as family conflict, may mediate these relationships and require further exploration (Sigfusdottir et al., 2017).

With respect to the association between AST's and conduct and emotional problems, the identified sex difference may reflect sex differences in autism traits more generally, i.e. autistic girls experience fewer conduct and social problems (Mandy et al., 2012) which in turn have a lesser effect on the relationship between hyperactive/inattentive traits and conduct and emotional difficulties.

## Clinical implications

The findings from this study should be used to inform the assessment, management and treatment strategies provided by educational and mental health services. Of particular importance is the accurate and timely identification of hyperactive/inattentive traits and ASTs, which can have a significant effect on behavioural and emotional outcomes across childhood and adolescence. There are two comprehensive and helpful resources available in open access publications providing clinical guidance and practical advice on the clinical approach for the care of people with co-occurring ADHD and autism (Young et al., 2020a; Young et al., 2020b).

The findings from this study also provide evidence for the need to assess for the presence of ASTs, particularly in boys, in order to more effectively manage and treat their difficulties. The identified sex differences may strongly contribute to the different rates of referrals for boys and girls with either ADHD, autism or both.

In addition, the more severe conduct and emotional problems experienced by boys may mask other neurodevelopmental symptoms and result in misdiagnoses, leading to inappropriate or ineffective interventions and support. In other words, young boys may be diagnosed with ADHD and conduct disorder and underlying autism or ASTs may be missed.

**Table:** 

Figure key	
Below Group	Below clinical threshold on the SCQ (⩽ 7)
Above Group	Above clinical threshold on the SCQ (⩾ 8)
ICEPT	Intercept
SLOPE	Slope
QUAD	Quadratic
CP	Conduct Problems
EP	Emotional Problems
H/I	Hyperactivity/Inattention
E	Error
→	Regression weights

Girls may benefit more from management strategies and treatment approaches that target symptoms of ADHD as opposed to ASTs in order to support their conduct and emotional problems. These findings further emphasise the need to consider the co-occurrence of ADHD and autism traits when working clinically with young people who are presenting with ADHD or autism in isolation. In addition, given the shared features and associated co-occurring conditions, further consideration should be given to the development of transdiagnostic approaches to treat and manage these conditions. The findings from the current study also offer some weight to the value of utilising a dimensional rather than a categorical approach when working with young people.

Given changes in conduct and emotional problems at different times for both boys and girls with ADHD, and boys with co-occurring ASTs, regular reviews should be conducted at key periods of personal transition, for example, transitions to secondary school and/or leaving school. Reviews should involve a collaborative approach with the young person, their family, neurodevelopmental services, Child and Adolescent Mental Health Services (CAMHS) and education professionals. In addition, it is important to raise awareness and training for CAMHS practitioners, educators and educational services about these findings in order to support their preparation and provisions for young people.

## Strengths and limitations

Strengths of the current study include the large general population sample, the inclusion of comparable numbers of boys and girls, and measurements at four time points, two-three years apart. Previous studies have often been limited to much smaller clinical samples, with disproportionately higher ratios of males, in order to explore the relationship between ADHD traits, conduct and emotional problems. Furthermore, a dimensional approach to all conditions removed any limitation that might be presented by using a categorical approach, for example the fact that more boys are diagnosed with ADHD and autism than girls (Fombonne, 2003; Gaub & Carlson, 1997), would bias the sample and findings. In addition, very few studies have explored patterns of change in these relationships over time and have been limited to group comparisons or two-wave designs. We acknowledge however that clinical studies have the advantage of investigating patients with severe presentations which is informative for healthcare practitioners in clinical practice.

Regarding limitations, firstly it should be noted that the SDQ was completed by parents. Therefore, perceived conduct and emotional problems may diverge from the child's actual experiences or the degree to which these experiences may impact on their functioning at home or alternative environments, such as school (Eiser & Morse, 2001; Staller & Faraone, 2006; Van der Meer, Dixon, & Rose, 2008; Van Roy, Groholt, Heyerdahl, & Clench-Aas, 2010). Secondly, only social communication difficulties were included in the multi-group analysis. It may be that the inclusion of restricted and repetitive behaviours and interests (core diagnostic features of autism) would have impacted on the observed variance. Thirdly, as a result of the SCDC cut off of eight (Skuse et al., 2009) the Above clinical threshold AST Group was comprised of far fewer young people than the Below clinical threshold AST Group and this may have had an impact on statistical power. Fourthly, the ASTs were measured at Time 1 (age 9 years) and whilst ASTs have been identified to remain relatively stable over time (Pourcain et al., 2011), modelling ASTs at each time point may have been more accurate. The final measurement (Time 4) was taken when the young people were aged 16 years old. ADHD symptoms are known to change across the lifespan (Barkley, Fischer, Smallish, & Fletcher, 2002; Faraone, Biederman, & Mick, 2006; Kooij, Buitelaar, Furer, Rijnders, & Hodiamont, 2005) and the time points selected for this study do not provide insight into the impact of ADHD symptoms and the moderating effect of ASTs on conduct and emotional problems into adulthood.

Future work should attend to the following limitations; whilst our approach accommodates potential shifts in hyperactive/inattentive symptoms overtime by treating hyperactive/inattentive symptoms as a time-variant covariate, we did not model the trajectory of hyperactivity/inattention change. Therefore, it is not possible to determine whether the time points used reflect progressive changes in symptoms or random fluctuations. In addition, because a univariate growth model, rather than a multivariate latent trajectory model was used, it is not possible to determine whether conduct and emotional problems are associated with changes in the trajectory of hyperactive/inattentive traits. Future research should consider whether there is a bi-directional relationship between the trajectories of conduct and emotional problems and hyperactive/impulsive traits.

It is important to recognise that parental report and associated biases may (at least partially) account for these findings. Existing research has indicated that parents tend to over-report conduct problems in boys and the underreport conduct problems in girls (Van Roy et al., 2010; Webster-Stratton, 1996). This is likely to contribute to the under-identification and subsequent lack of treatment for girls (Rucklidge, 2010).

## Research implications

Future research could expand the age range included within this study to better understand the onset of the relationship between ADHD traits and conduct and emotional problems and the trajectories for males and females of these outcomes into late adolescence, adulthood and even older adulthood. Functional impairments resulting from these problems could also be addressed. In addition, the current time points (9 years – 16 years) are likely to cover the onset of puberty for the majority of young people in the study. Future research could take into account the role and effect that this key developmental period may have on fluctuations in the trajectories of conduct and emotional problems.

In addition, selective characteristics of autism and ADHD were applied, future research could include other autism symptoms, such as restricted and repetitive behaviours and interests, or ADHD symptoms, such as impulsivity. Future research could also take into account other mediating factors.

## Conclusion

This study provides new and important information on the trajectories of conduct and emotional problems over four time periods for young people in the general population. It goes some way to uncover the intrinsic relationship that hyperactive/inattentive traits have on the expression of both of these outcomes. It also provides us with a unique understanding of how ASTs moderate these relationships for boys, but not for girls. The findings emphasise the need for the proactive and accurate identification of hyperactive/inattentive traits for both boys and girls in order to support them in managing these difficulties and reducing subsequent emotional and behavioural problems throughout childhood, adolescence and beyond.

## Supplementary material

For supplementary material accompanying this paper visit https://doi.org/10.1017/S0033291722001416. click here to view supplementary material
