# Supplementary material for: Sex differences in conduct and emotional outcomes for young people with hyperactive/inattentive traits and social communication difficulties between 9 and 16 years of age: a growth curve analysis
Source: Psychol Med. 2022 Jul 29;53(10):4539–49. doi: 10.1017/S0033291722001416 (PMC10388317; doi:10.1017/S0033291722001416)
Supplement: Supplementary file 1 [file S0033291722001416sup001.docx]

Supplementary Material

*Means and Standard Deviations of Hyperactivity/Inattention and outcomes by time points for boys and girls*

|  | Boys *n*  (M, *SD*) | Girls *n*  (M, *SD*) |
| --- | --- | --- |
| Hyperactivity/Inattention Time 1 | 4074 (3.34, 2.37) | 3981 (2.56, 2.06) |
| Hyperactivity/Inattention Time 2 | 3667 (3.23, 2.34) | 3665 (2.33, 2.02) |
| Hyperactivity/Inattention Time 3 | 3519 (3.33, 2.33) | 3531 (2.50, 2.03) |
| Hyperactivity/Inattention Time 4 | 2743 (2.81, 2.21) | 2922 (2.29, 2.00) |
| Conduct Problems Time 1 | 4078 (1.37, 1.50) | 3985 (1.21, 1.35) |
| Conduct Problems Time 2 | 3674 (1.28, 1.51) | 3677 (1.14, 1.33) |
| Conduct Problems Time 3 | 3516 (1.29, 1.47) | 3535 (1.21, 1.38) |
| Conduct Problems Time 4 | 2736 (0.97, 1.33) | 2929 (1.07. 1.37) |
| Emotional Problems Time 1 | 4066 (1.39, 1,71) | 3979 (1.66, 1.82) |
| Emotional Problems Time 2 | 3665 (1.33, 1.68) | 3668 (1.61, 1.78) |
| Emotional Problems Time 3 | 3518 (1.24, 1.61) | 3535 (1.63, 1.78) |
| Emotional Problems Time 4 | 2736 (1.09, 1.55) | 2919 (1.87, 2.02) |

*Goodness-of-fit statistics for Linear and Quadratic Models*

|  | Linear Model | | | | | | Quadratic Model | | | | | | |
| --- | --- | --- | --- | --- | --- | --- | --- | --- | --- | --- | --- | --- | --- |
|  | χ^2^(*df*) | CFI | RMSEA | | | AIC | χ^2^(*df*) | | CFI | RMSEA | | | AIC |
| Conduct Problems  (N=9305) | 161.717* (5) | .983 | | .058 | 179.717 | | 29.800* (1) | .997 | | | .056 | 55.800 | |
| Emotional Problems  (N=9300) | 80.167* (5) | .990 | | .040 | 98.167 | | 2.329 (1) | 1.000 | | | .012 | 28.329 | |

**p*<.001

*Quadratic Models*: *Goodness-of-fit statistics for conduct problems (Boys = 4675 and Girls = 4630) and emotional problems by gender (Boys = 4671 and Girls = 4629)*

|  | Boys | | | | Girls | | | |
| --- | --- | --- | --- | --- | --- | --- | --- | --- |
|  | χ^2^ (*df*) | CFI | RMSEA | AIC | χ^2^ (*df*) | CFI | RMSEA | AIC |
| Conduct Problems | 280.078*  (13) | .982 | .066 | 342.078 | 205.659*  (13) | .984 | .057 | 267.659 |
| Emotional Problems | 76.079*  (13) | .995 | .032 | 138.079 | 85.285*  (13) | .993 | .035 | 147.285 |

* *p*<.001

*Standardised regression weights (*β*) for Below clinical threshold ASTs group and Above clinical threshold ASTs group for conduct problems (CP) and emotional problems (EP) for girls*

|  | Below group | | | Above group | | |
| --- | --- | --- | --- | --- | --- | --- |
|  | β | S.E | C.R | β | S.E | C.R |
| Conduct Problems |  | | |  | | |
| H/I Time 1 -> CP Time 1 | .205* | .009 | 21.975 | .148* | .012 | 12.687 |
| H/I Time 2 -> CP Time 2 | .227* | .008 | 27.092 | .205* | .010 | 20.925 |
| H/I Time 3 -> CP Time 3 | .249* | .009 | 28.965 | .183* | .010 | 18.609 |
| H/I Time 4 -> CP Time 4 | .282* | 0.11 | 24.808 | .189* | .013 | 14.539 |
| Emotional Problems |  | | |  | | |
| H/I Time 1 -> EP Time 1 | .274* | .037 | 7.429 | .252* | .035 | 7.152 |
| H/I Time 2 -> EP Time 2 | .272* | .033 | 8.206 | .238* | .030 | 7.934 |
| H/I Time 3 -> EP Time 3 | .304* | .036 | 8.508 | .255* | .033 | 7.765 |
| H/I Time 4 -> EP Time 4 | .310* | .046 | 6.740 | .290* | .038 | 7.672 |

**p*<.001
